# Vagal modulation of high mobility group box-1 protein mediates electroacupuncture-induced cardioprotection in ischemia-reperfusion injury

**DOI:** 10.1038/srep15503

**Published:** 2015-10-26

**Authors:** Juan Zhang, Yue Yong, Xing Li, Yu Hu, Jian Wang, Yong-qiang Wang, Wei Song, Wen-ting Chen, Jian Xie, Xue-mei Chen, Xin Lv, Li-li Hou, Ke Wang, Jia Zhou, Xiang-rui Wang, Jian-gang Song

**Affiliations:** 1 Department of Anesthesiology, Shuguang Hospital Affiliated to Shanghai University of Traditional Chinese Medicine, Shanghai 201203, China; 2 Department of Anesthesiology, Renji Hospital, School of Medicine, Shanghai Jiao Tong University, Shanghai 200127, China; 3 Laboratory of Integrative Medical Surgery Department, Shuguang Hospital Affiliated to Shanghai University of Traditional Chinese Medicine, Shanghai 201203, China; 4 Department of Cardiothoracic Surgery, Shuguang Hospital Affiliated to Shanghai University of Traditional Chinese Medicine, Shanghai 201203, China; 5 Department of Nursing, Shanghai Pneumology Hospital, School of Medicine, Tongji University, Shanghai 200433, China; 6 Department of Anesthesiology, Shanghai Pneumology Hospital, School of Medicine, Tongji University, Shanghai 200433, China; 7 Department of Anesthesiology, 452nd Hospital of the People’s Liberation Army, Chengdu 610061, China; 8 Department of Anesthesiology, The first Affiliated Hospital of Soochow University, Suzhou, 215006, China

## Abstract

Excessive release of high mobility group box-1 (HMGB1) protein from ischemic cardiomyocytes activates inflammatory cascades and enhances myocardial injury after reperfusion. Here we report evidence that electroacupuncture of mice at Neiguan acupoints can inhibit the up-regulation of cardiac HMGB1 following myocardial ischemia and attenuate the associated inflammatory responses and myocardial injury during reperfusion. These benefits of electroacupuncture were partially reversed by administering recombinant HMGB1 to the mice, and further potentiated by administering anti-HMGB1 antibody. Electroacupuncture-induced inhibition of HMGB1 release was markedly reduced by unilateral vagotomy or administration of nicotinic receptor antagonist, but not by chemical sympathectomy. The cholinesterase inhibitor neostigmine mimicked the effects of electroacupuncture on HMGB1 release and myocardial ischemia reperfusion injury. Culture experiments with isolated neonatal cardiomyocytes showed that acetylcholine, but not noradrenaline, inhibited hypoxia-induced release of HMGB1 via a α7nAchR-dependent pathway. These results suggest that electroacupuncture acts via the vagal nerve and its nicotinic receptor-mediated signaling to inhibit HMGB1 release from ischemic cardiomyocytes. This helps attenuate pro-inflammatory responses and myocardial injury during reperfusion.

Acupuncture is gaining experimental support as an effective complementary therapy for treating coronary heart disease, arrhythmia, angina pectoris, and myocardial infarction^1^,^2^,^3^. Preoperative electroacupuncture has been shown to reduce cardiac troponin I (cTnI) release in adult and pediatric patients undergoing cardiac surgery, as well as shorten their stay in the intensive care unit^4^,^5^. Preoperative electroacupuncture at the Neiguan (PC6) and Ximen (PC4) acupoints has been shown to reduce cTnI release in patients with coronary artery disease undergoing percutaneous coronary intervention, as well as protect them from postoperative myocardial injury^6^. In an animal model of myocardial ischemia-reperfusion injury (MIRI), electroacupuncture at PC6 led to significantly less myocardial enzyme release, less frequent and severe arrhythmias and smaller infarct size^7^,^8^,^9^.

How electroacupuncture exerts these protective effects is unclear. It is possible that it works via the autonomic nervous system, since this system is important for regulating cardiac function. For example, chronic, intermittent low-level transcutaneous electrical stimulation of the auricular branch of the vagus nerve improved left ventricular remodeling in dogs following myocardial ischemia; this stimulation worked by inhibiting expression of several proteins in left ventricle tissue: collagen I, collagen III, transforming growth factor β1, and matrix metallopeptidase 9^10^. Vagal nerve stimulation during acute myocardial infarction in rats protected against arrhythmias and prevented the loss of phosphorylated Cx43^11^.

Several studies suggest that electroacupuncture protects against MIRI by modulating the autonomic nervous system’s control of cardiac function. Vagotomy partially reversed electroacupuncture-induced reduction in cardiac enzyme levels, arrhythmia duration and mortality rate in a rat model of MIR^8^. Electroacupuncture appears to exert cardioprotective effects in a rabbit model of MIRI at least in part by inhibiting cardiac norepinephrine release, thereby inhibiting the cardiac sympathetic nervous system^9^.

Despite these advances in mechanistic understanding, how electroacupuncture acts via the autonomic nervous system to induce cardioprotection remains unclear. Some have suggested that electroacupuncture pretreatment stimulates sympathetic activity, inducing the desensitization of β-adrenergic receptors via a mechanism similar to that of ischemic preconditioning^12^,^13^. Further studies are needed to examine whether and how electroacupuncture modulates autonomic nerve system activity in order to provide cardioprotection in MIRI.

One possibility is that electroacupuncture acts via the autonomic nervous system to inhibit the release of high mobility group box-1 (HMGB1) protein from ischemic myocardium. HMGB1 is a chromatin structural protein that is ubiquitously expressed and is up-regulated in response to myocardial ischemia^14^,^15^. Upon reperfusion, extracellular HMGB1 functions as a damage-associated molecular pattern (DAMP) molecule or danger signal, locally activating inflammatory cascades in ischemic tissue by binding to the receptor for advanced glycation end-products (RAGE) on bone marrow-derived macrophages. The influx of inflammatory cells into the stressed heart is a major cause of injury^16^,^17^. Administering neutralizing anti-HMGB1 antibodies to rats before inducing MIRI inhibited the ischemia-induced up-regulation of HMGB1 and led to less severe inflammatory response and myocardial injury^14^. In this way, HMGB1 acts as an early mediator of inflammation and organ damage in MIRI, leading us to speculate that electroacupuncture might exert its cardioprotective effects by inhibiting the ischemia-induced release of HMGB1.

In support of this hypothesis, vagal nerve stimulation in a rat model of sepsis was found to reduce HMGB1 release from activated macrophages, and the stimulation acted mainly via the neurotransmitter acetylcholine^18^. Conversely, autonomic nervous system dysfunction, observed as slow recovery of heart rate after exercise, was associated with elevated HMGB1 in patients after acute myocardial infarction^19^. Therefore we undertook the present study to examine whether electroacupuncture pretreatment in a mouse model of MIRI inhibits HMGB1 release from ischemic myocardium. This would help explain how electroacupuncture can significantly attenuate inflammatory cascades and myocardial injury during reperfusion.

## Results

### Electroacupuncture inhibits ischemia-induced expression of cardiac HMGB1 for up to 24 h of reperfusion

Ischemia has been shown to up-regulate cardiac HMGB1 expression and trigger its release so that it can act as a proinflammatory signal during reperfusion. To determine whether electroacupuncture may exert its cardioprotective effects by inhibiting this up-regulation, we subjected mice to ischemia-reperfusion with or without electroacupuncture pretreatment, and then analyzed expression of HMGB1 protein and mRNA as well as HMGB1 protein localization.

**Figure 1:**
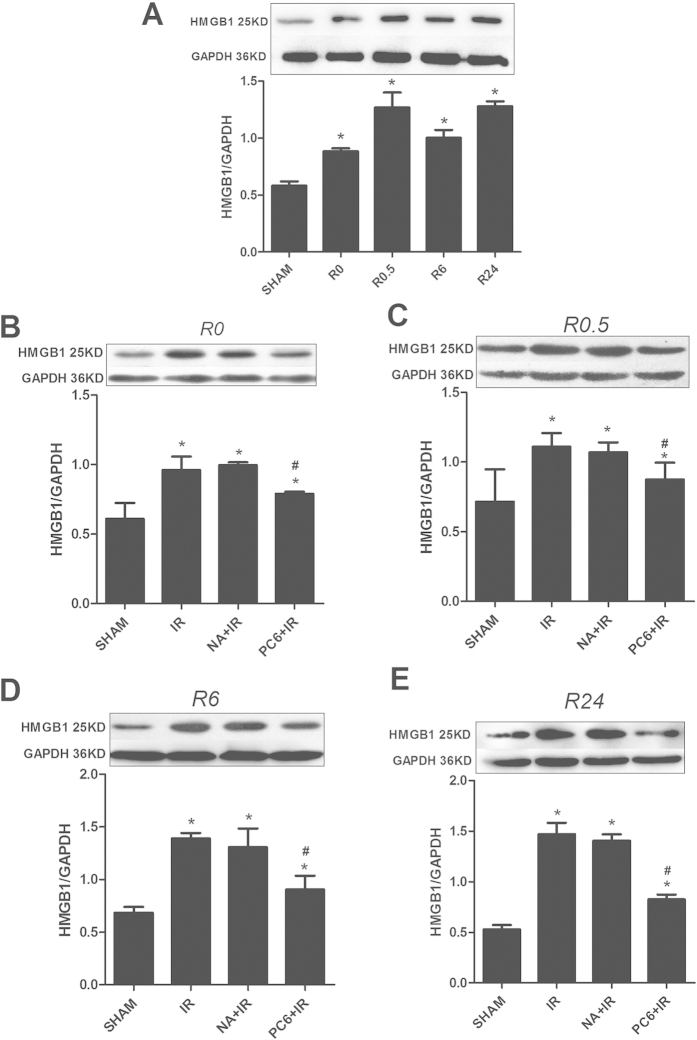
Electroacupuncture at Neiguan (PC6) acupoints reduces the up-regulation of HMGB1 protein induced by MIRI. Mice were subjected to 30 min of ischemia and sacrificed after 0, 0.5, 6 or 24 h of reperfusion (R). Prior to ischemia, mice were pretreated with electroacupuncture at PC6 acupoints (PC6+IR), electroacupuncture at non-acupoints (NA+IR), or no electroacupuncture (IR). Sham animals were mock-operated but not subjected to ischemia. (**A**) Levels of HMGB1 in left ventricle tissue at different time points of reperfusion were assessed by Western blot (n = 4). (**B**–**E**) Representative results and densitometric analysis at different time points of reperfusion are shown (n = 4). Data are mean ± SEM. *p < 0.05 vs. Sham, ^#^*p* < 0.05 vs. IR (one-way ANOVA followed by Bonferroni correction).

**Figure 2:**
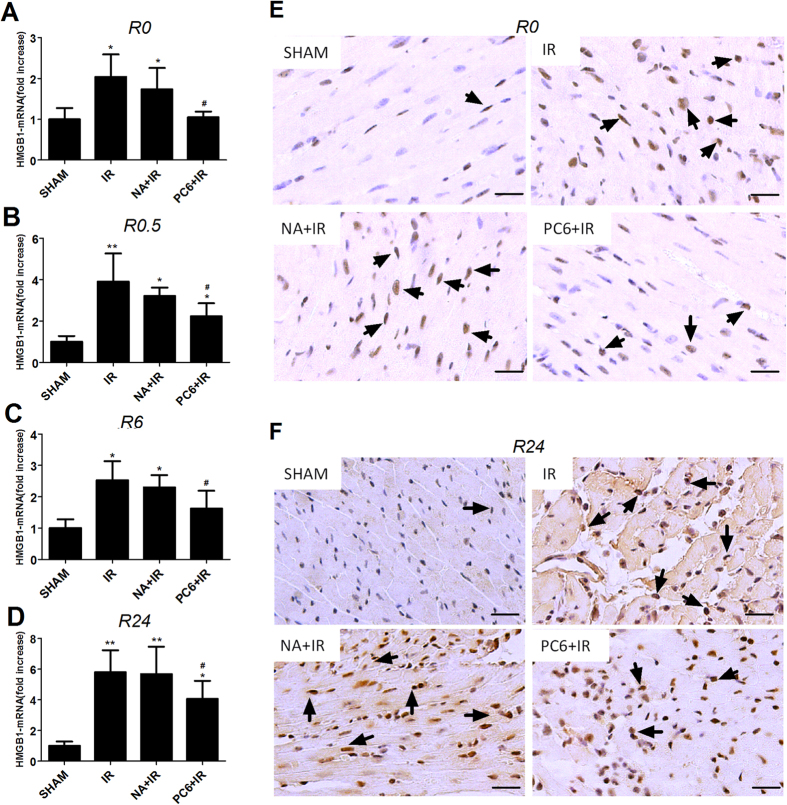
Electroacupuncture at Neiguan (PC6) acupoints reduces the up-regulation of HMGB1 mRNA and increase in HMGB1-positive cells induced by MIRI. Experiments were carried out as described in Fig. 1(A–D) Levels of HMGB1 mRNA in the different treatment groups are shown relative to the levels in the Sham group (n = 4). (**E**,**F**) Representative images of immunohistochemical staining for HMGB1 immediately after ischemia (R0) and after 24 h of reperfusion (R24) are shown (n = 3). Arrows indicate HMGB1-positive cells. Scale bar, 50 μm. Data are mean ± SEM. *p < 0.05 vs. Sham; **p < 0.01 vs. Sham; ^#^*p* < 0.05 vs. IR(A-D: one-way ANOVA followed by Bonferroni correction).

Western blot analysis of left ventricle lysates showed higher HMGB1 levels in ischemia-reperfusion animals than in sham-treated controls immediately after ischemia as well as after 0.5, 6 and 24 h of reperfusion (all p < 0.05; Fig. 1A). Electroacupuncture pretreatment at PC6 acupoints, but not at non-acupoints, inhibited this ischemia-induced increase in myocardial HMGB1 expression at all time points, at the level of both protein (PC6+IR vs. IR, p < 0.05, Fig. 1B–E) and mRNA (PC6+IR vs. IR, p < 0.05, Fig. 2A–D).

Similar results were obtained using HMGB1 immunohistochemistry in myocardial sections. Immediately after ischemia (time point R0), the number of HMGB1-positive cells was much higher in ischemia-reperfusion animals than in sham-treated animals. HMGB1 was expressed mainly in the nucleus of cardiomyocytes (Supplementary Figure 2). Electroacupuncture pretreatment at PC6 acupoints, but not at non-acupoints, decreased the number of HMGB1-positive cells in ischemia-reperfusion animals, although it remained higher than the number in control animals (Fig. 2E). After 24 h of reperfusion (time point R24), levels of HMGB1 protein in the nucleus, cytoplasm and interstitial spaces were higher than in animals not subjected to ischemia-reperfusion (Fig. 2F). Electroacupuncture pretreatment at PC6 acupoints, but not at non-acupoints, partially blocked these increases (Fig. 2F).

These results suggest that electroacupuncture may exert its cardioprotective effects by inhibiting ischemia-induced up-regulation of HMGB1. Next we examined how this inhibition may mediate cardioprotection.

### Electroacupuncture inhibits HMGB1-mediated inflammatory cascades and helps prevent myocardial injury following ischemia-reperfusion

**Figure 3:**
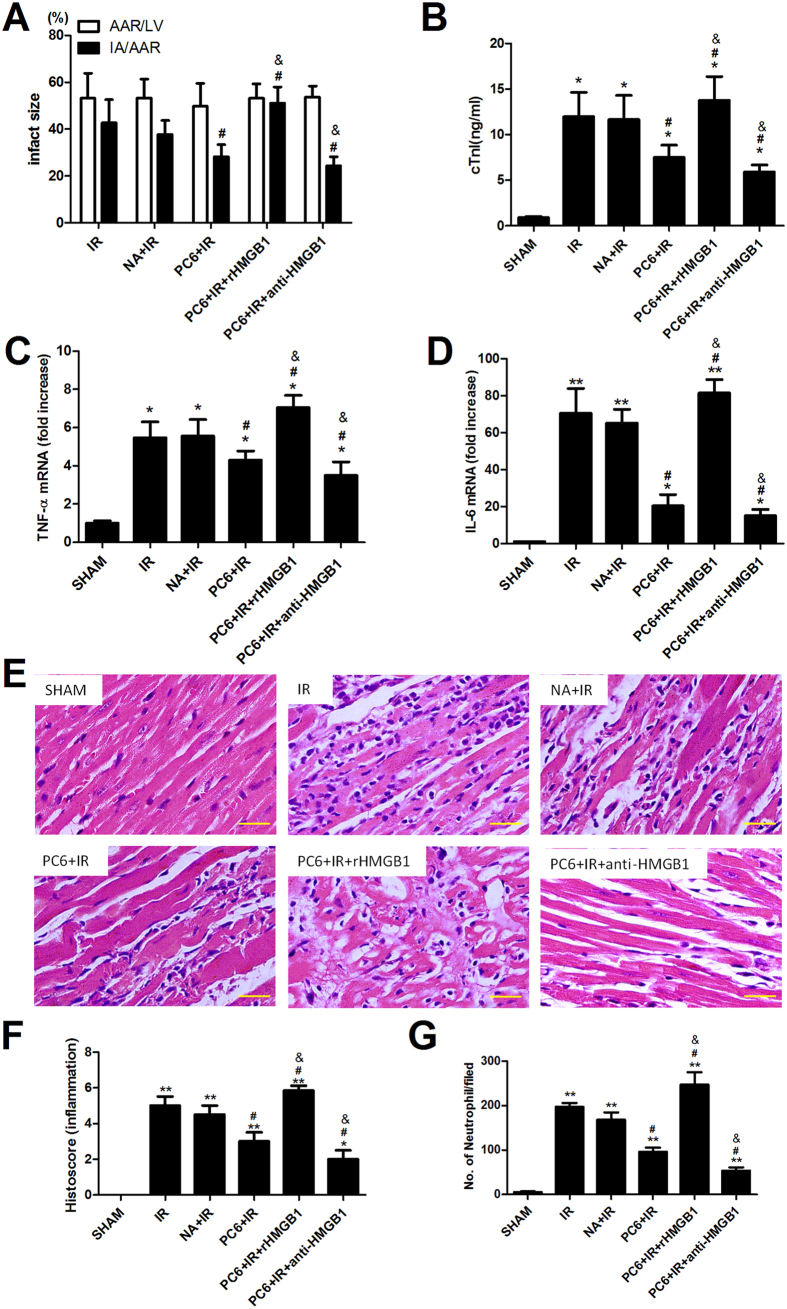
Cardioprotective effects of electroacupuncture after ischemia-reperfusion involve inhibition of HMGB1-mediated inflammatory cascades and tissue injury. Mice were subjected to 30 min of ischemia and sacrificed after 24 h of reperfusion (R). Prior to ischemia, mice were pretreated with electroacupuncture at PC6 acupoints (PC6+IR), electroacupuncture at non-acupoints (NA+IR), or no electroacupuncture (IR). Sham animals were mock-operated but not subjected to ischemia. Two additional groups were pretreated with recombinant HMGB1 (PC6+IR+rHMGB1) or with anti-HMGB1 neutralizing antibody (PC6+IR +anti-HMGB1) prior to electroacupuncture at PC6 acupoints and ischemia-reperfusion. (**A**) Infarct size in each group was calculated as IA/AAR%, and AAR/LV% was presented to show the reproducibility of the mouse MIRI model (n = 10). (**B**) Serum levels of cardiac troponin I were measured by ELISA (n = 6). (**C**,**D**) Myocardial levels of TNF-α and IL-6 mRNA were measured relative to levels in Sham animals (n = 6). (**E**,**F**) Representative images of sections stained with hematoxylin-eosin are shown together with average histopathology scores (n = 4 for each group). (**G**) Neutrophil infiltration in myocardium was quantified per field (n = 6 for each group; original magnification ×40). Scale bar, 100 μm. Data are mean ±  SEM. *p < 0.05 vs. Sham; **p < 0.01 vs. Sham; ^#^*p* < 0.05 vs. IR; ^&^*p* < 0.05 vs. PC6+IR. (Histology scores of injury severity were compared using the Kruskal-Wallis rank test and Mann-Whitney U test. All other data were analyzed using one-way ANOVA followed by Bonferroni correction).

To confirm the validity of our mouse model for examining how electroacupuncture may protect against MIRI, we subjected mice to ischemia-reperfusion with or without electroacupuncture pretreatment, then compared the two groups in terms of infarct size, serum levels of cardiac troponin I, levels of the myocardial inflammatory mediators TNF-α and IL-6, extent of neutrophil infiltration in myocardium and histopathology score. At 24 h of reperfusion, infarct size was significantly smaller in mice pretreated with electroacupuncture at PC6 acupoints than in mice without electroacupuncture treatment (28.15 ± 5.02% vs. 42.64 ± 9.48%, p = 0.029; Fig. 3A). Infarct size was similar in mice pretreated with electroacupuncture at non-acupoints and in mice without electroacupuncture treatment (37.67 ± 5.44% vs. 42.64 ± 9.48%, p > 0.05).

At the same time point, electroacupuncture pretreatment at PC6 acupoints led to significantly lower serum levels of cardiac troponin I than no pretreatment (PC6+IR vs. IR, p = 0.042; Fig. 3B). Electroacupuncture treatment at non-acupoints gave similar results as no pretreatment (NA+IR vs. IR, p > 0.05). Ischemia-reperfusion led to significantly higher levels of TNF-α (IR vs. Sham, p = 0.035; Fig. 3C) and IL-6 (IR vs. Sham, p < 0.01; Fig. 3D), and these increases were significantly smaller after electroacupuncture pretreatment at PC6 acupoints (PC6+IR vs. IR, p = 0.043 for TNF-α and p = 0.021 for IL-6).

Ischemia-reperfusion led to significant histopathology in cardiac sections stained with hematoxylin-eosin, which was visible as necrosis, hypereosinophilia, loss of nuclei and of transverse striation, as well as neutrophil infiltration (Fig. 3E–G). The average histopathological score for cardiac tissue after ischemia and 24 h of reperfusion was 4.5 ± 0.5. Electroacupuncture pretreatment at PC6 acupoints significantly reduced visible tissue damage and inflammatory infiltration (Fig. 3E,F), as well as neutrophil infiltration in myocardium (Fig. 3G). As a result, the average histopathological score was 2.5 ± 0.5.

The experiments described above suggest that electroacupuncture can act through HMGB1 to protect against MIRI. To corroborate these findings using more direct methods, we subjected mice to ischemia-reperfusion with or without electroacupuncture pretreatment in the presence or absence of exogenous recombinant HMGB1 (rHMGB1) or anti-HMGB1 neutralizing antibody. Administration of rHMGB1 significantly attenuated the cardioprotective and anti-inflammatory effects of electroacupuncture pretreatment. Conversely, administration of anti-HMGB1 antibody enhanced electroacupuncture-elicited decreases in infarct size; serum levels of cardiac troponin I, TNF-α, and IL-6; histopathology score; and neutrophil infiltration (Fig. 3A–G).

These results suggest that our mouse model accurately captures key aspects of ischemia-reperfusion injury, and that electroacupuncture pretreatment exerts its cardioprotective effects, at least in part, through HMGB1. Next we examined whether these HMGB1-mediated effects involve the autonomic nervous system, since studies have suggested that electroacupuncture acts via the autonomic system.

### Electroacupuncture-dependent myocardial protection and inhibition of HMGB1 release depends on the vagal nerve, but not the sympathetic nerve

To examine which autonomic neuronal pathways may mediate the cardioprotective effects of electroacupuncture, we subjected mice to surgical unilateral vagotomy or chemical sympathectomy, followed by electroacupuncture or not, and finally ischemia-reperfusion. We compared levels of myocardial HMGB1 expression and indices of ischemia-reperfusion injury between groups immediately after ischemia and after 24 h of reperfusion.

**Figure 4:**
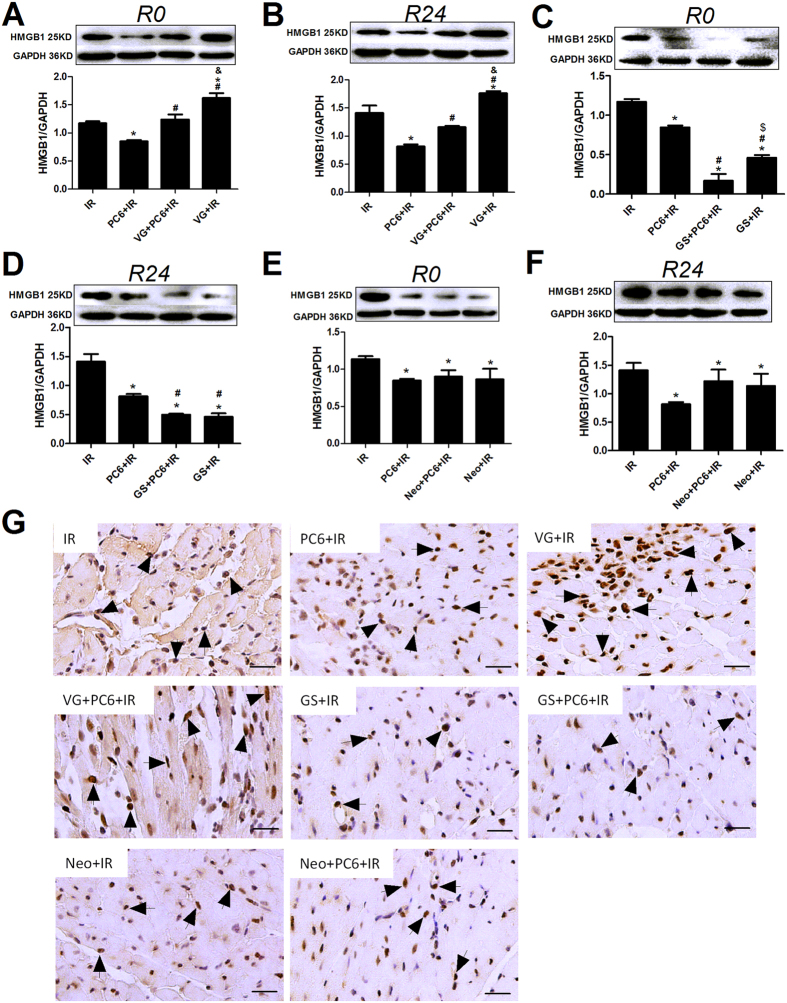
The ability of electroacupuncture to inhibit myocardial HMGB1 release after ischemia-reperfusion depends on the vagal nerve, but not the sympathetic nerve. Mice were subjected to 30 min of ischemia and sacrificed after 0 and 24 h of reperfusion. Prior to ischemia, mice were pretreated as described in Fig. 2, except that groups were subjected to unilateral vagotomy (VG), chemical sympathectomy by injection with guanethidine sulfate (GS) or vagal stimulation by injection with neostigmine (Neo). (**A**–**F**) Levels of HMGB1 protein were assessed in left ventricle tissue by Western blot (n = 4). (G) Representative images of tissue after 24 h of reperfusion and immunostaining for HMGB1 are shown (n = 3). Arrows indicate HMGB1-positive cells. Scale bar, 50 μm. Data are mean ±  SEM. *p < 0.05 vs. IR; ^#^*p* < 0.05 vs. PC6+IR; ^&^*p* < 0.05 vs. VG+PC6+IR; ^$^*p* < 0.05 vs. GS+PC6+IR (**A–F**: one-way ANOVA followed by Bonferroni correction).

**Figure 5:**
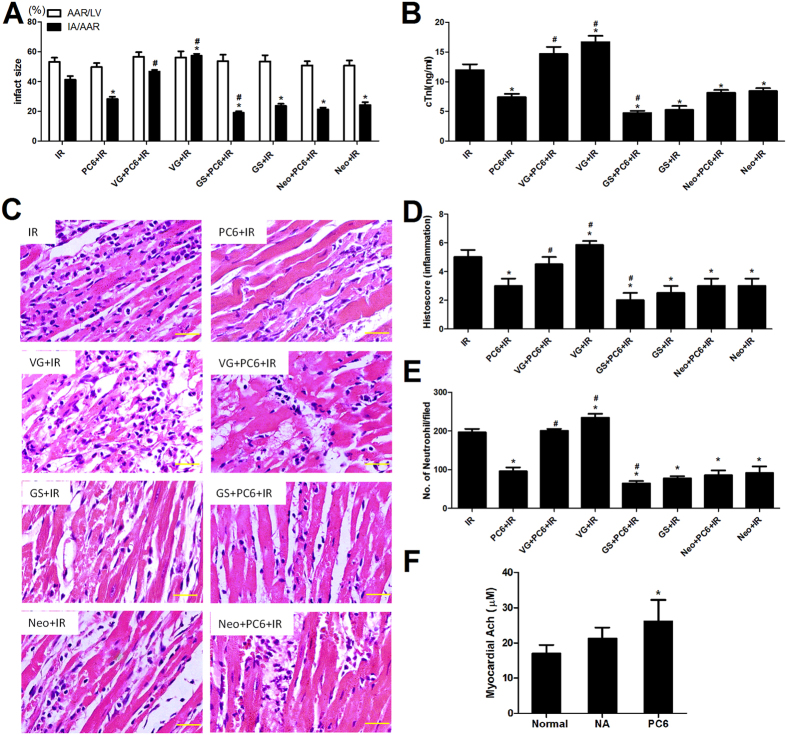
The ability of electroacupuncture to protect the myocardium after ischemia-reperfusion depends on the vagal nerve, but not the sympathetic nerve. Mice were treated as described in Fig. 4, except that animals were sacrificed only after 24 h of reperfusion. (**A**) Infarct size in each group was calculated as IA/AAR%, and AAR/LV% was determined to show the reproducibility of the mouse MIRI model (n = 10). (**B**) Serum levels of cardiac troponin I were measured by ELISA (n = 10). (**C**,**D**) Representative images of tissue after staining with hematoxylin-eosin are shown together with average histopathology scores (n = 4). Neutrophil infiltration in myocardium was quantified per field (n = 6 for each group; original magnification, ×40). Scale bar, 100 μm. Data are mean ±  SEM. *p < 0.05 vs. IR; ^#^*p* < 0.05 vs. PC6+IR. (Histology scores of injury severity were compared using the Kruskal-Wallis rank test and Mann-Whitney U test. All other data were analyzed using one-way ANOVA followed by Bonferroni correction.) (**F**) Levels of myocardial acetylcholine (Ach) were measured in healthy animals with or without electroacupuncture without subsequent ischemia-reperfusion (n = 6). Data are mean ± SEM. *p < 0.05 vs. Normal (one-way ANOVA followed by Bonferroni correction).

Among animals not pretreated by electroacupuncture, HMGB1 expression was higher in animals subjected to unilateral vagotomy than in sham-operated animals both immediately after ischemia (VG+IR vs. IR, p = 0.032; Fig. 4A) and after 24 h of reperfusion (p = 0.035; Fig. 4B). Vagotomized animals also showed larger infarct size than sham-operated ones (VG+IR vs. IR, p = 0.037; Fig. 5A), as well as greater release of cardiac troponin I (p = 0.040; Fig. 5B) and more severe histological damage and inflammation infiltration (p = 0.042; Fig. 5C–E).

Among animals pretreated by electroacupuncture at PC6 acupoints, HMGB1 release was significantly higher in animals subjected to unilateral vagotomy than in sham-operated animals, both immediately after ischemia (VG+PC6+IR vs. PC6+IR, p = 0.024; Fig. 4A,B) and after 24 h of reperfusion (p = 0.030). Similarly, cardioprotective effects of electroacupuncture were less notable in animals subjected to unilateral vagotomy (Fig. 5A–E).

Conversely to the effects of vagotomy, chemical sympathectomy before ischemia-reperfusion led to lower HMGB1 expression than in animals not subjected to sympathectomy (GS+IR vs. IR, p < 0.05; Fig. 4C,D), as well as less severe myocardial injury (Fig. 5A–E). The combination of chemical sympathectomy and electroacupuncture pretreatment inhibited HMGB1 expression immediately after ischemia to a greater extent than did electroacupuncture alone (GS+PC6+IR vs. PC6+IR, p = 0.023; Fig. 4C), and it also provided greater cardiac protection (p < 0.05, Fig. 5A–E).

These results suggest that the vagal nerve may mediate the ability of electroacupuncture to inhibit HMGB1 release and its cardioprotective effects during MIRI. As a further test of this hypothesis, we repeated the experiments with or without pretreatment with neostigmine, which increases vagal tone. Pretreating mice with neostigmine and then subjecting them to ischemia-reperfusion without electroacupuncture led to less HMGB1 release than simply subjecting the animals to ischemia-reperfusion (Neo+IR vs. IR, p < 0.05; Fig. 4E,F), and it also led to less severe myocardial injury (p < 0.05; Fig. 5A–E). In fact, neostigmine pretreatment gave results similar to those with electroacupuncture pretreatment (Neo+IR vs. PC6+IR, p > 0.05; Figs 4 and 5). We did not see evidence of an additive effect, as pretreatment with both electroacupuncture and neostigmine led to similar levels of HMGB1 expression as electroacupuncture alone (Neo+PC6+IR vs. PC6+IR, p > 0.05; Fig. 4E,F), as well as similar cardioprotection (Fig. 5A–E).

These results suggest that electroacupuncture, like neostigmine, works through the vagal nerve to inhibit the release of HMGB1 from ischemic cardiomyocytes. If electroacupuncture works by stimulating the vagal nerve, we would expect electroacupuncture to increase myocardial levels of acetylcholine. As predicted, mice treated with electroacupuncture at PC6 acupoints (without subsequent ischemia-reperfusion) showed higher levels of myocardial acetylcholine than untreated mice (Fig. 5F). In contrast, mice treated with electroacupuncture at non-acupoints showed similar acetylcholine levels as untreated mice.

### Blockade of nAchRs and α7nAchRs, but not mAchRs, abolishes electroacupuncture-induced inhibition of HMGB1 release and cardioprotection during MIRI

After obtaining evidence that electroacupuncture works by stimulating the vagal nerve, we attempted to identify which types of acetylcholine receptor may be involved. We repeated the ischemia-reperfusion experiments with or without pretreatment with the muscarinic acetylcholine receptor (mAchR) antagonist atropine, the nicotinic acetylcholine receptor (nAchR) antagonist mecamylamine, or the selective α7nAChR antagonist methyllycaconitine.

**Figure 6:**
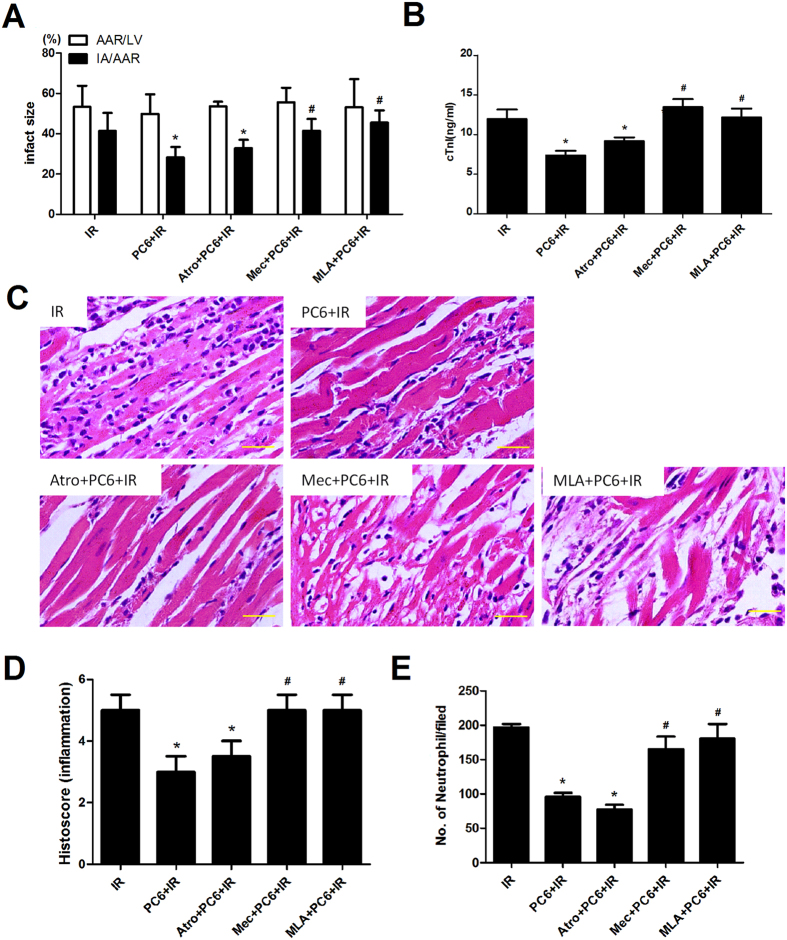
Antagonists of nAchR and α7-nAchR, but not of mAchR, abolish cardioprotective effects of electroacupuncture against MIRI. Atropine (Atro), mecamylamine (Mec) and methyllycaconitine (MLA) were administered to mice that were later subjected to electroacupuncture and ischemia-reperfusion. Animals were sacrificed and examined after 24 h of reperfusion. (**A**) Infarct size in each group was calculated as IA/AAR%, and AAR/LV% was determined to show reproducibility of the mouse MIRI model (n = 10). (**B**) Serum levels of cardiac troponin I were measured by ELISA (n = 10). (**C**,**D**) Representative images of tissue stained with hematoxylin-eosin are shown together with average histopathology scores (n = 4). (**E**) Neutrophil infiltration in myocardium was quantified per field (n = 6 for each group; original magnification, ×40). Scale bar, 100 μm. Data are mean ±  SEM. *p < 0.05 vs. IR; ^#^*p* < 0.05 vs. PC6+IR. (Histology scores of injury severity were compared using the Kruskal-Wallis rank test and Mann-Whitney U test. All other data were analyzed using one-way ANOVA followed by Bonferroni correction.) All data come from 3 independent experiments.

**Figure 7:**
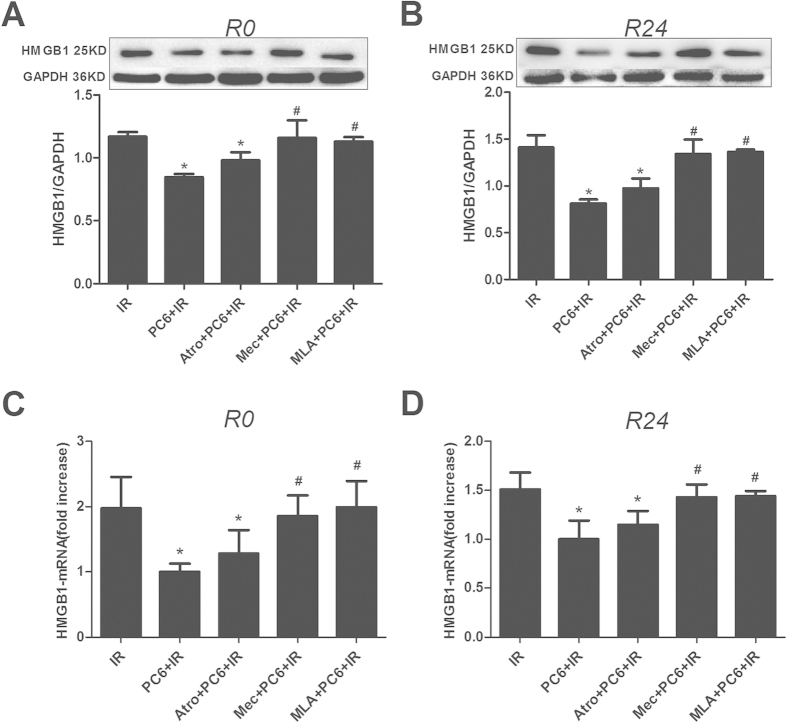
Antagonists of nAchR and α7nAchR, but not of mAchR, abolish electroacupuncture-induced inhibition of myocardial HMGB1 expression after ischemia-reperfusion. Atropine (Atro), mecamylamine (Mec) and methyllycaconitine (MLA) were administered to mice that were later subjected to electroacupuncture and ischemia-reperfusion. Animals were sacrificed and examined after 0 and 24 h of reperfusion. (**A**,**B**) Levels of HMGB1 protein in left ventricle tissue were determined by Western blot. Representative results and densitometric analysis are shown (n = 4). (**C**,**D**) Levels of HMGB1 mRNA in left ventricle tissue were determined by quantitative PCR. Representative results are shown with respect to PC6+IR (n = 4). Data are mean ±  SEM. *p < 0.05 vs. IR; ^#^*p* < 0.05 vs. PC6+IR (one-way ANOVA followed by Bonferroni correction). All data come from 3 independent experiments.

Both mecamylamine and methyllycaconitine partially reversed the cardioprotective effects of electroacupuncture (Mec+PC6+IR or MLA+PC6+IR vs. PC6+IR, p < 0.05; Fig. 6A–E), while atropine did not significantly affect them (Atro+PC6+IR vs. PC6+IR, p > 0.05; Fig. 6A–E). Similar results were obtained for HMGB1 expression: co-pretreatment with electroacupuncture and either mecamylamine or methyllycaconitine before ischemia-reperfusion led to similar levels of HMGB1 mRNA and protein as no pretreatment (Mec+PC6+IR or MLA+PC6+IR vs. IR; p > 0.05, Fig. 7A–D). Pretreatment with atropine, however, did not affect the ability of electroacupuncture to inhibit myocardial HMGB1 expression after ischemia (Atro+PC6+IR vs. PC6+IR, p > 0.05; Fig. 7A–D).

These results suggest that the nicotinic acetylcholine receptor (nAchR), especially the α7nAChR, but not the muscarinic acetylcholine receptor (mAchR), are likely to mediate the effects of electroacupuncture during MIRI.

### Acetylcholine, but not noradrenaline, inhibits hypoxia-induced HMGB1 release from neonatal cardiomyocytes *in vitro*

**Figure 8:**
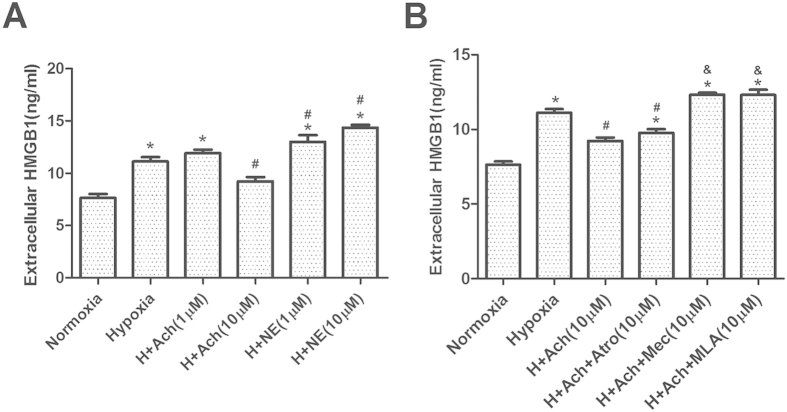
Acetylcholine, but not noradrenaline, inhibits hypoxia-induced release of HMGB1 from primary cardiomyocytes, which depends on nAchR, primarily α7nAchR. Neonatal rat cardiomyocytes were cultured under hypoxic conditions for 6 h in the presence of different concentrations of acetylcholine (Ach) or noradrenaline (NE). (**A**) Levels of HMGB1 in the culture medium were determined by ELISA (n = 4). (**B**) Experiments were repeated by co-administering 10 μM acetylcholine with atropine (Atro), mecamylamine (Mec) or methyllycaconitine (MLA) (n = 4). Data are mean ±  SEM. *p < 0.05 vs. Normoxia; ^#^*p* < 0.05 vs. Hypoxia; ^&^*p* < 0.05 vs. H+Ach (one-way ANOVA followed by Bonferroni correction). All data come from at least 3 independent experiments.

To provide a complementary *in vitro* system for testing the hypothesis that the autonomic nervous system may help mediate ischemia-induced release of HMGB1 from cardiomyocytes, we examined the effects of hypoxia on neonatal cardiomyocyte cultures in the presence or absence of acetylcholine or noradrenaline. Treating cells with 10 μM acetylcholine during hypoxia led to much lower levels of HMGB1 in the extracellular medium than hypoxia alone [H+Ach (10 μM) vs. Hypoxia, p = 0.039; Fig. 8A]. In contrast, treating cells with noradrenaline did not significantly affect the hypoxia-induced increase in HMGB1 release [H+NE (1 or 10 μM) vs Hypoxia, p < 0.05; Fig. 8A]. These results are consistent with the activity of electroacupuncture in the *in vivo* mouse model of MIRI.

### Acetylcholine-induced inhibition of HMGB1 release during hypoxia in neonatal cardiomyocytes *in vitro* depends on nAchR, primarily α7nAchR

To complement the *in vivo* findings implicating nAchRs in electroacupuncture’s mechanism of action, we repeated the cell culture experiments by co-administering acetylcholine with atropine, mecamylamine or methyllycaconitine during hypoxia. Mecamylamine and methyllycaconitine abolished the ability of acetylcholine to inhibit HMGB1 release (H+Ach+Mec or H+Ach+MLA vs. H+Ach, p < 0.05; Fig. 8B), but atropine did not (H+Ach+Atro vs. H+Ach, p > 0.05; Fig. 8B). These results provide complementary support for the idea that electroacupuncture protects against MIRI by stimulating nAchRs.

## Discussion

Using an *in vivo* mouse model of MIRI, we provide evidence that electroacupuncture pretreatment reduces inflammation and protects cardiac tissue following reperfusion by inhibiting HMGB1 expression and release from ischemic cardiomyocytes. These effects require vagal nerve transmission through nicotinic receptors, but not sympathetic nerve transmission. The cholinesterase inhibitor neostigmine, which increases cardiac vagal tone, mimics the ability of electroacupuncture to inhibit HMGB1 release and protect cardiac tissue against MIRI. We complemented these *in vivo* insights by subjecting neonatal cardiomyocyte cultures to hypoxia. Hypoxia triggered release of HMGB1 into the culture medium, and this release was inhibited by acetylcholine, but not noradrenaline. This effect of acetylcholine depended on nAchRs, primarily α7nAchRs.

Sterile injury activates the innate immune system to cause cytokine and chemokine production patterns quite similar to those produced during infection. Excessive activation can lead to severe local and systemic inflammation that exacerbates tissue damage^20^. Results from our laboratory^21^ and from other groups^22^ have shown that electroacupuncture can prevent excessive systemic inflammatory responses that normally arise after lipopolysaccharide injection or cecal ligation and puncture. As a result, electroacupuncture enhances survival after these insults. The present study extends this finding by showing that electroacupuncture also inhibits inflammatory cascades and concomitant tissue injury induced by sterile injury (e.g. MIRI).

Our results suggest that electroacupuncture exerts its anti-inflammatory and cardioprotective effects by inhibiting HMGB1 release from cardiomyocytes. For example, pretreating mice with exogenous recombinant HMGB1 prior to ischemia-reperfusion weakened the beneficial effects of electroacupuncture, while pretreating them with anti-HMGB1 antibody potentiated the beneficial effects. HMGB1 is an alarmin, like heat shock proteins, S100s, and hyaluronan; these proteins, normally intracellular, are released passively by necrotic cells or actively secreted by stressed cells in response to injury^23^. In this way, HMGB1 and other alarmins notify the immune system of damage. During cardiac ischemia, HMGB1 is released from cardiomyocytes and binds to RAGE, and this binding promotes an early inflammatory response that amplifies the initial inflammation, ultimately exacerbating myocardial injury^14^.

While our findings are consistent with the idea that electroacupuncture directly influences the expression and activity of myocardial HMGB1, we cannot exclude the possibility that electroacupuncture acts at another site, subsequently causing the observed effects in the myocardium. This is because our experiments involving intraperitoneal injection of anti-HMGB1 neutralizing antibody neutralizing antibody or exogenous HMGB1 do not affect cardiac tissue exclusively. Nevertheless, we feel confident that electroacupuncture directly affects the myocardium, since this tissue showed the most severe inflammation and change in HMGB1 expression after MIRI. Future studies should verify our findings using cardio-specific loss or gain of HMGB1 function.

HMGB1 was first identified as a late-stage cytokine-like mediator of lethality in sepsis models, and the vagal nerve neurotransmitter acetylcholine was found to inhibit the active secretion of HMGB1 from human macrophages in a α7nAchR-dependent process^18^. Recently, HMGB1 was found to function as a damage-associated molecular pattern (DAMP) molecule or danger signal as well. Evidence from our *in vivo* mouse model of MIRI was corroborated by *in vitro* studies, which showed that acetylcholine, but not norepinephrine, inhibits hypoxia-induced HMGB1 release from isolated neonatal cardiomyocytes through a process dependent on nAchRs, primarily α7nAchRs. This is an important advance because it shows that acetylcholine-based vagal neurotransmission can inhibit HMGB1 release from either immune cells or parenchymal cells such as cardiomyocytes in response to sterile injury.

Our findings are consistent with previous work suggesting that electroacupuncture activates the vagal nerve. Electroacupuncture at PC6 acupoints led to significantly higher normalized high-frequency power of heart rate variability than sham acupuncture^24^. Electroacupuncture has been shown to inhibit the release of cardiac norepinephrine during MIRI^25^, which provides indirect evidence that electroacupuncture increases vagal tone, given that vagal nerve stimulation can inhibit sympathetic nerve activity through pre- and post-synaptic processes in the peripheral nervous system^26^,^27^. Our results also provide direct evidence that electroacupuncture at PC6 acupoints, but not at non-acupoints, significantly increases myocardial levels of acetylcholine. Future studies should examine whether electroacupuncture-induced vagal tone stimulation involves activation of central muscarinic receptors^21^ or inhibition of cardiovascular premotor sympathetic neuronal discharge in the rostral ventrolateral medulla^28^,^29^.

The demonstration that electroacupuncture can modulate HMGB1 expression through the vagal nerve provides new therapeutic possibilities for treating pathological inflammation. Targeting HMGB1 itself may prove challenging because alarmins, like many signaling molecules, exert both harmful and beneficial effects within the same disease context, depending on dose and timing of release. For example, although HMGB1 triggers detrimental inflammation soon after certain injuries, it functions later as a repair molecule, mediating myocardial regeneration after myocardial infarction by inducing resident cardiac c-kit^+^ progenitor cell proliferation and differentiation. This helps repair the injured tissue and restore cardiac function^30^, as well as promote the migration and growth of endothelial progenitor cells and their organization into tubules^31^. HMGB1 can attenuate mitochondrial dysfunction and apoptosis during doxorubicin-induced cardiomyopathy^32^, and administration of exogenous HMGB1 at 4 h after myocardial infarction improves cardiac function^33^. Targeting HMGB1 would require careful timing so as to activate the innate protective pathways that initiate regenerative and anti-apoptotic processes while down-regulating the self-injurious pathways that inhibit repair and drive excessive cytokine release. It may prove easier to develop therapeutic approaches that target electroacupuncture-induced vagal modulation of HMGB1 release.

## Methods

### Animals

Male C57BL6 mice aged 8–10 weeks were obtained from the Sino-British SIPPR/BK Lab Animal Ltd. Co. (Shanghai, China). All animals received standard diet and water ad libitum and were treated in accordance with the Guide for the Care and Use of Laboratory Animals prepared by the Institute of Laboratory Animal Research at the US National Institutes of Health. The study protocol was approved by the Animal Care Committee of Shanghai Jiao Tong University (Shanghai, China).

### Mouse model of MIRI

All mice were subjected to MIRI as described^34^. Briefly, mice were anesthetized with 1–2% isoflurane and ventilated with a TOPO rodent ventilator (Kent Scientific). Adequacy of anesthesia was checked using a tail pinch test. Lateral thoracotomy at the fourth intercostal space was performed by blunt dissection of the intercostal muscles following skin incision. The left anterior descending artery was ligated using a 6–0 silk suture. Thoracic wall incisions were sutured with 6.0 non-absorbable silk sutures, and the skin wound was closed using skin adhesive. Ligation was maintained for 30 min, after which reperfusion was established by loosening the knot. Sham animals underwent the same surgery as animals subjected to ischemia-reperfusion, except that the coronary artery was not occluded.

### Electroacupuncture pretreatment

At 1 h prior to ischemia induction, electroacupuncture was delivered to Neiguan acupoints (PC6) on both forelimbs; this acupoint is located between the radius and ulna on the ventral surface of the forelimb at 1 mm from the wrist^35^. As a control, other animals were treated by electroacupuncture at non-acupoints (NA) at 1 mm above the elbow in the midline of the dorsal surface, where acupoints are sparse. The needles were inserted to a depth of 3 mm and held in place with plastic adhesive tape. Stimulation was delivered using an electrical stimulation device (HANS LH-202, Huawei, China). The 30-min stimulation consisted of the following alternating dense-and-disperse currents (1 mA): 2 Hz (0.6-ms pulse width) and 100 Hz (0.2-ms pulse width), each of which lasted for 3 s^36^. Surgery prior to occlusion of the left anterior descending artery was performed within 30 min of the end of electroacupuncture stimulation, as this time window has been shown to be most effective^8^).

### Pretreatment with recombinant HMGB1 and anti-HMGB1 antibody

Mice received single intraperitoneal injections of recombinant human HMGB1 (10 μg per mouse; Sigma-Aldrich)^14^ or anti-HMGB1 neutralizing polyclonal antibody (600 μg per mouse; Shino-Test Corporation)^37^ at 1 h before ischemia induction, concurrent with the start of electroacupuncture pretreatment. Negative control mice were injected with saline instead of rhHMGB1 or with non-immune IgG (Sigma Aldrich) instead of anti-HMGB1 antibody.

### Pretreatment with surgical vagotomy or chemical sympathectomy

To investigate the involvement of the vagal and sympathetic nerves, mice were subjected to unilateral vagotomy or chemical sympathectomy prior to electroacupuncture pretreatment and ischemia-reperfusion. Unilateral vagotomy was carried out as described^38^. Briefly, the cervical vagal nerve on the right side of anesthetized mice was ligated with a 5–0 silk suture and transected under sterile conditions. Mice were allowed to recover for 4 days prior to experiments in order to avoid nerve stimulation during surgery^38^. Sham animals underwent the same surgery as animals subjected to vagotomy, except that the cervical vagal nerve was not transected. Sympathectomy was achieved with a single subcutaneous injection of guanethidine sulfate (30 mg/kg; Santa Cruz Biotechnology) at 1 h prior to electroacupuncture and ischemia-reperfusion^39^.

### Other pretreatments

The effect of vagal outflow was potentiated by intraperitoneal injection of neostigmine (80 μg/kg; Sigma Aldrich)^40^ at 30 min before ischemia-reperfusion. In other experiments, the possible involvement of specific types of acetylcholine receptors was examined. At 30 min before ischemia-reperfusion, mice received a single injection of the mAchR antagonist atropine sulfate (1 mg/kg, i.p.;^41^), nAChR antagonist mecamylamine hydrochloride (1 mg/kg, s.c.^42^) or the α7-nicotinic acetylcholine receptor antagonist methyllycaconitine(1mg/kg,s.c.^42^). All drugs were purchased from Sigma-Aldrich.

### Infarct size determination

Mice were pretreated in different ways and then subjected to 30 min of ischemia, followed by 24 h of reperfusion. Then infarct size was evaluated using Evans blue and triphenyltetrazolium chloride staining (TTC) (Sigma-Aldrich), as described^43^. Briefly, the left anterior descending coronary artery was ligated at 24 h after reperfusion, followed by retrograde injection of 2% Evans blue into the aortic arch. The heart was sliced into five sections of equal thickness perpendicular to the long axis, incubated in 1% TTC for 15 min at 37 °C and fixed in 10% formalin overnight. Images were captured using a microscope (DFC500, Leica, Germany) equipped with a digital camera (C-DSD230, Nikon, Japan). The left ventricle (LV) area, area at risk (AAR) and infarct area (IA) were determined using ImageJ (U.S. National Institutes of Health) and adjusted for weight. An example with Evans blue and TTC staining showing the delineation of infarct boundaries was shown in Supplementary Figure 1. AAR/LV% and IA/AAR% were calculated using the following formulas:


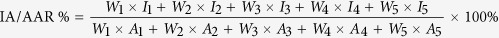



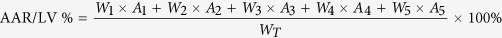


where *W*_*n*_ represents the weight of each heart section; *W*_*T*_, the weight of the entire heart; *I*_*n*_, percentage of infarct area (IA) with white color on each section; and *A*_*n*_, the percentage of area at risk (AAR) with white and red color on each section.

### Western blot analysis of HMGB1 levels

Mice were pretreated in different ways and then subjected to 30 min of ischemia, followed by reperfusion for different periods. At the indicated time points during reperfusion, mice were sacrificed by bleeding and the heart was explanted under general anesthesia (1–2% isoflurane). Left ventricles were frozen in liquid nitrogen and stored at −80 °C until analysis. Ventricle samples were homogenized in radioimmunoprecipitation assay buffer (RIPA buffer) containing protease inhibitor cocktail (Roche). The homogenate was centrifuged for 15 min at 12,000 *g* at 4 °C. An aliquot of supernatant (40 μg total protein) was fractionated on 12% SDS-polyacrylamide gels and transferred onto nitrocellulose membranes. Membranes were blocked with 5% nonfat dry milk and 0.01% Tween-20 in Tris-buffered saline (TBS) for 1 h, then incubated overnight at 4 °C with primary monoclonal antibody against HMGB1 (1:1000; Abcam) or GAPDH (1:10000; Kangcheng, China). Finally the membranes were incubated with an anti-rabbit secondary antibody conjugated to horseradish peroxidase (HRP) (Biotime, China), and the membranes were processed using chemiluminescent HRP substrate (Millipore). Bands were visualized and quantitated using a ChemiDoc^TM^ XRS+ system (Bio-Rad).

### Real-time PCR analysis of mRNA levels of HMGB1 and inflammatory markers

At the indicated time points after ischemia-reperfusion following different pretreatments, animals were sacrificed and total RNA was extracted from the left ventricle using Trizol (Invitrogen). Real-time PCR analysis was performed using a real-time PCR kit (Takara Bio) and specific primers to amplify regions coding HMGB1 (5′-TTTAGATAGCCCTGTCCTGGTGGTA-3′ reverse, 5′-GTGCACCAACAAGAACCTGCTTTA-3′), interleukin-6 (5′-CCACTTCACAAGTCGGAGGCTTA-3′; 5′-GCAAGTGCATCATCGTTGTTCATAC-3′), tumor necrosis factor-α (5′-CAGGAGGGAGAACAGAAACTCCA-3′; 5′-GCCTACTCATTGGGATCATCTTG-3′), GAPDH as a control (5′-GGTTGTCTCCTGCGACTTC-3′; 5′-CCTGTTGCTGTAGCCGTATTCAT-3′).

### Histology to assess inflammation severity

Under deep anesthesia animals were laparotomized and perfused with heparin-PBS followed by 10% formalin. The left ventricle was removed and paraffin-embedded. Every fifth section (4 μm) was stained with hematoxylin-eosin (HE). An investigator blinded to treatment condition assessed the extent of inflammation as described^44^ using the following scale: grade 0, no inflammation; grade 1, cardiac infiltration in up to 5% of cardiac sections; grade 2, 6–10%; grade 3, 11–30%; grade 4, 31–50%; grade 5, 51–70%; grade 6, >70%. Six fields were picked from each section, and 5 sections were examined for each animal; the scores were then averaged. Using a similar approach, neutrophil infiltration was quantitated by counting neutrophils. Six fields were picked from each section, and 5 sections were examined for each animal; the numbers were then averaged.

### Immunohistochemistry to assess HMGB1 levels

Left ventricle sections were prepared as described for histology and incubated with a primary antibody against mouse HMGB1 (1:100; Abcam), followed by a biotin-conjugated secondary antibody and finally with avidin-peroxidase (ABC Kit; Vector Laboratories). The reaction was developed using the DAB substrate kit (Vector Laboratories). Sections were then counterstained with hematoxylin and analyzed on a BX-51 microscope (Olympus, Japan) using ImageJ software.

### ELISA assays of cardiac troponin I and acetylcholine

Serum levels of cardiac troponin I were assayed using an ELISA kit (Life Diagnostics). Acetylcholine levels in the myocardium were assayed by homogenizing the left ventricle on ice and measuring neurotransmitter concentration using the Amplex Red Acetylcholine/Acetylcholinesterase Assay Kit (Invitrogen). All analyses were performed according to the manufacturers’ instructions.

### Hypoxia-induced changes in rat cardiomyocytes *in vitro*

Cardiomyocytes were isolated from neonatal rats at 1–3d after birth and cultured as described^45^. Briefly, rat heart was harvested, minced and digested with collagenase II (1 mg/ml; Sigma Aldrich). Cells were isolated by centrifugation through Percoll gradients (Sigma-Aldrich), preplated for 2 h and then finally seeded into 35-mm culture dishes (3 × 10^5^/ml). Cells were cultured until they achieved 95% confluence and showed synchronous contraction, typically after 72 h, at which point they were used in subsequent experiments.

To investigate whether acetylcholine or noradrenaline can directly affect hypoxia-induced release of HMGB1, we treated cells with either neurotransmitter at 1 or 10 μM or with vehicle medium (DMEM/F12 without glucose and serum). Cells were cultured under hypoxic conditions for 6 h at 37 °C in an atmosphere of 1% O_2_, 5% CO_2_ and balanced N_2_ in a NUNC incubator (Wiesbaden, Germany). To explore the potential involvement of acetylcholine receptors in cardiomyocyte response to hypoxia, we treated the cells for 30 min before hypoxia and throughout hypoxia with a combination of acetylcholine (10 μM) and the mAchR antagonist atropine (10 μM), nAchR antagonist mecamylamine (10 μM) or parenchymal α7nAchR antagonist methyllycaconitine (10 μM). HMGB1 in the culture medium was assayed using an ELISA kit (IBL International).

### Statistics

Data are expressed as mean ± SEM and were analyzed using PRISM software (GraphPadPrism Institute). Results for numerical variables were compared using one-way ANOVA, followed by Bonferroni correction for multiple comparisons. Histology scores of injury severity were compared using the Kruskal-Wallis rank test and Mann-Whitney U test. The threshold for significance was set at p < 0.05.

## Additional Information

**How to cite this article**: Zhang, J. *et al*. Vagal modulation of high mobility group box-1 protein mediates electroacupuncture-induced cardioprotection in ischemia-reperfusion injury. *Sci. Rep*. **5**, 15503; doi: 10.1038/srep15503 (2015).

## Supplementary Material

Supplementary Information
