# Supplementary material for: Vagal modulation of high mobility group box-1 protein mediates electroacupuncture-induced cardioprotection in ischemia-reperfusion injury
Source: Sci Rep. 2015 Oct 26;5:15503. doi: 10.1038/srep15503 (PMC4620449; doi:10.1038/srep15503)

Supplementary information:

**Vagal modulation of high mobility group box-1 protein mediates electroacupuncture-induced cardioprotection in ischemia-reperfusion injury**

Juan Zhang^2,8†^, Yue Yong^3†^, Xing Li^3^, Yu Hu^7^, Jian Wang^1^, Yong-qiang Wang^1^, Wei Song^1^, Wen-ting Chen^1^, Xue-mei Chen^2^, Xin Lv^6^, Li-li Hou^5^, Ke Wang^3^, Jia Zhou^4^, Xiang-rui Wang^2^, Jian-gang Song^1^*

1. Department of Anesthesiology, Shuguang Hospital Affiliated to Shanghai University of Traditional Chinese Medicine, Shanghai 201203, China

2. Department of Anesthesiology, Renji Hospital, School of Medicine, Shanghai Jiao Tong University, Shanghai 200127, China

3. Laboratory of Integrative Medical Surgery Department, Shuguang Hospital Affiliated to Shanghai University of Traditional Chinese Medicine, Shanghai 201203, China

4. Department of Cardiothoracic Surgery, Shuguang Hospital Affiliated to Shanghai University of Traditional Chinese Medicine, Shanghai 201203, China

5. Department of Nursing, Shanghai Pneumology Hospital, School of Medicine, Tongji University, Shanghai 200433, China

6. Department of Anesthesiology, Shanghai Pneumology Hospital, School of Medicine, Tongji University, Shanghai 200433, China

7. Department of Anesthesiology, 452nd Hospital of the People’s Liberation Army, Chengdu 610061, China

8. Department of Anesthesiology, The first Affiliated Hospital of Soochow University, Suzhou, 215006, China

^†^Both authors contributed equally.

* Corresponding author: Jian-Gang Song, e-mail: [songjg1993@126.com](mailto:邮箱songjg1993@126.com); Tel: +86-(0)21-20256302; Fax: +86-(0)21-20256302;

Department of Anesthesiology, Shuguang Hospital Affiliated to Shanghai University of Traditional Chinese Medicine, 528 Zhanghe Road, Shanghai 201203, China


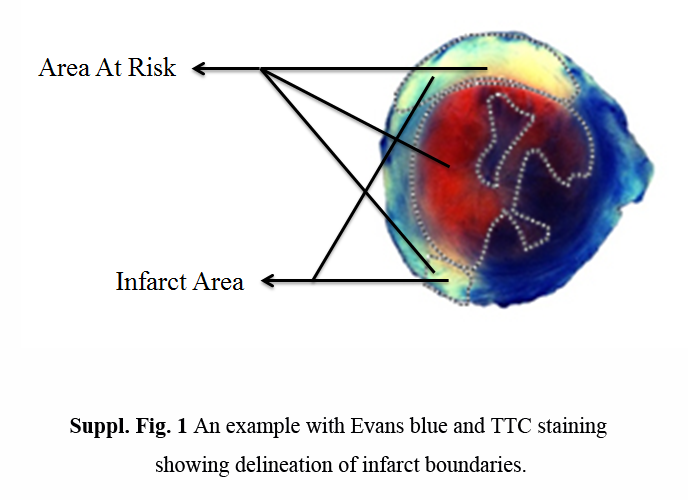


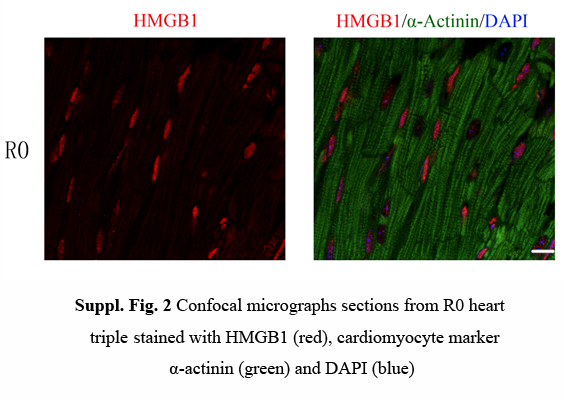

Supplement: Supplementary Information [file srep15503-s1.doc]
